# Factors associated with mental health outcomes among caregivers of older adults in long-term care facilities during COVID-19 post-epidemic era in Shandong, China

**DOI:** 10.3389/fpsyt.2022.1011775

**Published:** 2022-10-12

**Authors:** Huiling Chen, Yingjuan Cao, Yanxia Lu, Xiaolei Zheng, Bin Kong, Hua Dong, Qingbo Zhou

**Affiliations:** ^1^Department of Geriatrics, The Second Hospital, Cheeloo College of Medicine, Shandong University, Jinan, China; ^2^Department of Nursing, Qilu Hospital, Cheeloo College of Medicine, Shandong University, Jinan, China; ^3^School of Nursing and Rehabilitation, Cheeloo College of Medicine, Shandong University, Jinan, China; ^4^Nursing Theory and Practice Innovation Research Center, Cheeloo College of Medicine, Shandong University, Jinan, China; ^5^Department of Medical Psychology and Ethics, School of Basic Medical Sciences, Cheeloo College of Medicine, Shandong University, Jinan, China; ^6^Department of Neurology, The Second Hospital, Cheeloo College of Medicine, Shandong University, Jinan, China; ^7^Department of Pension Service, Jinan Civil Affairs Bureau, Jinan, China; ^8^Department of Secretariat, Jinan Pension Service Development Promotion Association, Jinan, China

**Keywords:** COVID-19, long-term care facility, nursing home, mental health, caregivers of older adults

## Abstract

**Background:**

COVID-19 pandemic has altered the work mode in long-term care facilities (LTCFs), but little is known about the mental health status of caregivers of older adults.

**Methods:**

A total of 672 formal caregivers of older adults in LTCFs and 1,140 formal patient caregivers in hospitals (comparison group) responded to an online survey conducted from March 25, 2022 to April 6, 2022. Five psychological scales, including Insomnia Severity Index (ISI), Generalized Anxiety Disorder-7 (GAD-7), Patient Health Questionnaire-9 (PHQ-9), The 5-item World Health Organization Wellbeing Index (WHO-5) and Perceived Stress Scale-14 item (PSS-14), were applied to assess participants’ mental health status. Factors, including sex, profession, marital status, economic conditions, length of working experience, frequent night shift beyond 1 day per week and having organic diseases, were included in logistic regression analysis to identify associated factors with mental health outcomes of formal caregivers of older adults in LTCFs.

**Results:**

Caregivers of older adults in LTCFs developed similar severe psychological symptoms with patient caregivers in hospital setting. For caregivers of older adults in LTCFs, unmarried status was a potent risk factor for insomnia, anxiety, impaired wellbeing and health risk stress, with odds ratios ranging from 1.91 to 3.64. Frequent night shift beyond 1 day per week was associated with higher risks of insomnia, depression and impaired wellbeing. Likewise, having organic disease or inferior economic condition, and being nurses appeared to be independent predictors for multiple mental health-related outcomes.

**Conclusion:**

During COVID-19 post-epidemic era, caregivers of older adults in LTCFs had a higher prevalence of psychological symptoms, especially those with particular risk factors. Special attention should be paid to promote their mental health.

## Introduction

Since the outbreak of coronavirus disease 2019 (COVID-19) in Wuhan, China at the end of December 2019 (1), the world has witnessed multiple waves of a global pandemic as new coronavirus variants continued to emerge. By June 2020, China had effectively brought the epidemic under control and declared a new stage of regular epidemic prevention and control during the post-pandemic era (2). Since then, the COVID-19 cases in China had been controlled in a low level. However, the more contagious and concealed coronavirus variant, Omicron, first emerged in late November 2021, in South Africa (3), and swiftly spread into China setting off an unprecedented epidemic peak since March 2022. Given the severity of the epidemic abroad and concrete national conditions, the Chinese government adopt strict prevention and control interventions such as mass confinement, social isolation, increased sanitation, and quarantine measures (4).

According to the 2020 population census of China (5), the national population aged 65 and above accounted for 13.5%. Shandong province has 15.36 million people aged 65 and above and the proportion reached 15.1%, making it the province with the largest population over the age of 65 in China. Due to the rapid aging of society, more and more long-term care facilities (LTCFs) for older adults, such as hospital operated older adult care institutions, traditional nursing homes and older adult apartments (6), have been equipped with high-quality medical care under the joint efforts of the National Health Commission and the Ministry of Civil Affairs since 2015 (7). Shandong is one of the demonstration provinces of the combination of medical and nursing care in China, and its development of medical and nursing care of older adults is relatively more mature. These LTCFs for older adults can accommodate the older adults who is disabled, demented, in the convalescent stages of disease or needs hospice care services (8), so higher requirements are put forward for caregivers in these institutions. As the older adults are the vulnerable, high-risk populations to COVID-19, LTCFs are particularly dangerous places regarding the spread of COVID-19 (9, 10). Since early March 2022, at the early stage of the unprecedented epidemic peak caused by Omicron variant, most LTCFs in China implemented strict closed management measures (i.e., all staff should live and work in LTCFs all the time and be barred from any direct contact with outside world) following the guidance of the Ministry of Civil Affairs for a second time (the first time was at the beginning of 2020) (11).

The caregivers of older adults, consisting of nurses and nursing assistants, are the main front-line care workers in LTCFs for older adults, thus they are at high risk of developing a mental illness because of the heavy health care burden and the long-term dependence of disabled older adults (12–14). Researches had shown that symptoms of insomnia, anxiety, depression, lower wellbeing and stress were the main challenges faced by caregivers of older adults (12, 14–17). During the COVID-19 epidemic period, the strict closed measures and uncertain release time are very probable to aggravate their mental health problems (18). Nevertheless, to date there is rare research published focused on the mental health problems of the caregivers of older adults in this strict closed management setting in China. Many studies have focused on the mental health status of populations such as healthcare workers and non-healthcare workers, medical and non-medical students, nurses, and general public (19–22). We posit that the prevalence of psychological symptoms in LTCFs caregivers of older adults is not much lower than that of patient caregivers in hospitals not admitting COVID-19 patients. However, to our knowledge, no research has investigated mental health levels of caregivers of older adults in LTCFs and patient caregivers in hospitals at the same period during COVID-19 post-epidemic era in China.

To address this gap, the aim of the present study was to assess the mental health status of LTCFs caregivers of older adults in strict closed management setting, analyze the potential risk factors and compared their mental health levels with patient caregivers in hospitals at the early stage of the unprecedented epidemic peak caused by Omicron variant. For this purpose, the prevalence and potential factor of insomnia, anxiety, depression, wellbeing and stress were detected.

## Materials and methods

### Design, participants, and procedure

This is a cross-sectional study using convenience sampling method conducted via an online survey from March 25, 2022 to April 6, 2022. This period corresponded to the early stage of the unprecedented epidemic peak caused by Omicron variant, much higher than the COVID-19 epidemic peak 2 years ago in Wuhan (1). The survey began 8 days after all LTCFs in Shandong Province implemented strict closed management measures on March 17, 2022, as required by Department of Civil Affairs of Shandong Province. At the same period, non-LCTF institutions, including hospitals, strengthened the frequency of COVID-19 detection but were not under closed management. Data were collected through Wenjuanxing platform^1^ with an anonymous, self-rated questionnaire. Since the sample size should be 5–10 times the number of scale items and considering a sample dispersion rate of 20%, it was expected that at least 638 participants would be required in each group.

The inclusion criteria were as follows: (a) for the caregivers of older adults’ group, participant was a front-line formal caregivers of older adults working in a LTCF; (b) for the patient caregivers’ group, participant was a front-line formal patient caregiver working in a hospital; (c) participant’ workplace was in Shandong province; and (d) participant volunteered to take part in this study. The exclusion criteria were as follows: (a) staff who had been suffered from a diagnosed mental illness by a doctor before the COVID-19 epidemic; (b) workers who were retired or in maternity or sick leave; (c) non-front-line caregivers such as administrative and logistics staff in LTCFs or hospitals; and (d) questionnaire response time was less than 120 s.

From all 16 cities of Shandong Province, 8 cities (Jinan, Qingdao, Zibo, Weihai, Dongying, Heze, Taian, Binzhou) with large and well-managed nursing homes were selected to ensure sample size of caregivers of older adults’ group and patient caregivers’ group. All institutions selected in study possessed their own WeChat work group and applet with a questionnaire was sent to these WeChat groups. The questionnaire included questions on sociodemographic characteristics and mental health assessment. All participants were informed of the purpose of the survey, aiming to better understand the mental health status associated with the COVID-19 epidemic, before responses. A simple slide puzzle was included at the end to ensure the quality and completeness of responses with the questionnaire. Subjects could quit the process at any time, so it ensures the participants were those who volunteered to complete the online questionnaire. Besides, the study was approved by the Medical Ethics Committee of the Second Hospital of Shandong University (No. KYLL-2022LW060).

After accounting for inclusion and exclusion criteria, a total of 1,945 online questionnaires were distributed, from which 1,812 valid questionnaires were collected after deleting the unqualified questionnaires. The effective response rate was 93.1%. The effective respondents of LTCFs caregivers of older adults’ group and hospitalized patients’ caregivers’ group were 672 and 1,140, and the effective response rates were 89.9 and 95.1%, respectively.

### Measurements

#### Sociodemographic data

Basic sociodemographic data include age, sex (male or female), profession (nursing assistants, nurses), institution (LTCFs, hospitals), marital status (married, unmarried, divorced, widowed), economic conditions (income < expenditure or income ≥ expenditure), education level (≤9 years or >9 years), length of working experience (≤3 years, 3–6 years, > 6 years), frequent night shift beyond 1 day per week (yes or no) and having organic diseases diagnosed by medical examination in hospital (yes or no). Subjects were also asked whether they have had mental health disorders diagnosed in hospital prior to COVID-19, and those who replied yes were excluded from the study.

#### Mental health assessment

A total of five Chinese versions of validated psychological scales with good psychometric properties were applied to assess symptoms of insomnia, anxiety and depression, wellbeing and stress (23–27).

#### Insomnia

Insomnia Severity Index (ISI) was a brief seven-item self-report questionnaire used to assess the severity of initial, middle and late insomnia; distress about sleep difficulties; interference of insomnia with daytime functioning and notice of sleep problems by others (28, 29). A 5-point Likert scale ranging from 0 to 4 was used to rate each item, yielding a total score ranging from 0 to 28 (30). The total score of ISI was interpreted as follows: normal (from 0 to 7), subthreshold (from 8 to 14), moderate (from 15 to 21), and severe (from 22 to 28) insomnia (23). The clinical cut-off score for detecting symptoms of insomnia was 15 (31). Cronbach’s alpha for Chinese version of ISI was 0.84.

#### Symptoms of anxiety

Generalized Anxiety Disorder-7 (GAD-7), a self-report questionnaire with a seven-item scale, was used to measure anxiety symptoms (32). GAD-7 was a 4-point scale ranging from 0 to 3 and the total score therefore ranges from 0 to 21 (33, 34). The total score of GAD-7 was interpreted as follows: normal (from 0 to 4), mild (from 5 to 9), moderate (from 10 to 13), moderate to severe (from 14 to 18), severe (from 19 to 21) anxiety symptoms (24). The clinical cut-off score for detecting symptoms of anxiety was 10 (35). Cronbach’s alpha for Chinese version of GAD-7 was 0.90.

#### Symptoms of depression

Patient Health Questionnaire-9 (PHQ-9) was used to assess depression symptoms experienced over the preceding 2 weeks (36). PHQ-9 is a self-report questionnaire with nine-item scale. Each item was rated by a 4-point scale ranging from 0 to 4 and the total score ranges from 0 to 27 (37). The total score of PHQ-9 was interpreted as follows: normal (from 0 to 4), mild (from 5 to 9), moderate (from 10 to 14), moderate to severe (from 15 to 19), severe (from 20 to 27) anxiety symptoms (25). The clinical cut-off score for detecting symptoms of anxiety was 10 (35). Cronbach’s alpha for Chinese version of PHQ-9 was 0.86.

#### Wellbeing

The 5-item World Health Organization Wellbeing Index (WHO-5) was applied to measure wellbeing (38). WHO-5 questionnaire measured wellbeing with five self-rating items rated on 6-point Likert scales with higher score indicating higher wellbeing (39). The total score therefore ranges from 0 (absence of wellbeing) to 25 (maximal wellbeing). The clinical cut-off point was set at 13 and a summed score below 13 indicated lower wellbeing or depression (26). Cronbach’s alpha for Chinese version of WHO-5 was 0.90.

#### Stress

Perceived Stress Scale-14 item (PSS-14), a fourteen-item self-rated scale, was used to measure perceived stress (40). A 5-point Likert-type scale ranging from 0 to 4 was used to rate each item and therefore the total score ranges from 0 to 56 (41, 42). The clinical cut-off point was set at 25 and a total score higher than 25 represented health risk stress (43). Cronbach’s alpha for Chinese version of PSS-14 is 0.78.

### Statistical analysis

Data analysis was performed using the SPSS statistical software version 25 (IBM Corp.). To compare the sociodemographic characteristics of LTCFs caregivers of older adults’ group with hospitalized patient caregivers’ group, Chi-square test for categorical variables and independent-sample non-parametric Mann-Whitney *U* test were used. The original scores of the 5 measurement tools were not normally distributed and thus were presented as medians with interquartile ranges (IQRs). The non-parametric Mann-Whitney U test was used to compare each symptom scores of LTCFs caregivers of older adults’ group with hospitalized patient caregivers’ group. Chi-square test was used to compare severity categories of each symptom measurements. Multivariate logistic regression analysis was performed using stepwise variable selection, and variables, including sex, profession, marital status, economic conditions, length of working experience, frequent night shift beyond 1 day per week and having organic diseases, were entered into each model to explore potential risk factors for symptoms of insomnia, anxiety, depression, wellbeing and stress among caregivers of older adults in LTCFs. The associations between risk factors and outcomes are presented as odds ratios (ORs) and 95% confidence intervals (CIs). The significance level was set at α = 0.05, and all tests were 2-tailed.

## Results

### Demographic characteristics

A total of 1,812 responders, including 672 caregivers of older adults in LTCFs and 1,140 patient caregivers in hospitals, completed the survey via WeChat applet. The sociodemographic characteristics of the three groups is shown in Table 1. Results of LTCF caregivers of older adults’ group showed that the mean age of caregivers of older adults in LTCFs (41.0 ± 12.4) was older than that of patient caregivers in hospitals (36.3 ± 9.0). Most of (85.0%) caregivers of older adults in LTCFs were female. More than half of (55.8%) caregivers of older adults in LTCFs were nursing assistants. The majority of (77.7%) caregivers of older adults in LTCFs were married. The proportion of formal educational level ≤9 years in LTCFs caregivers of older adults’ group (36.6%) was higher than hospitalized patient caregivers’ group (10.8%). For length of working experience, only 34.7% caregivers of older adults in LTCFs worked for more than 6 years. A large proportion of (71.6%) of caregivers of older adults in LTCFs had frequent night shift (>1 day per week).

**TABLE 1:** Sociodemographic characteristics of responders.

Characteristics	Total (*n* = 1,812)	Caregivers of older adults in LTCFs (*n* = 672)	Patient caregivers in hospitals (*n* = 1,140)	*P*-values*
Age (year)	38.0 ± 10.6	41.0 ± 12.4	36.3 ± 9.0	<0.01
Sex, No. (%)				<0.01
Male	207 (11.4)	101 (15.0)	106 (9.3)	
Female	1,605 (88.6)	571 (85.0)	1,034 (90.7)	
Profession, No. (%)				<0.01
Nursing assistants	545 (30.1)	375 (55.8)	170 (14.9)	
Nurses	1,267 (69.9)	297 (44.2)	970 (85.1)	
Marital status, No. (%)				<0.01
Unmarried	308 (17.0)	150 (22.3)	158 (13.9)	
Married^#^	1,504 (83.0)	522 (77.7)	982 (86.1)	
Economic conditions, No. (%)				0.26
Income < expenditure	319 (17.6)	127 (18.9)	192 (16.8)	
Income ≥ expenditure	1,493 (82.4)	545 (81.1)	948 (83.2)	
Formal educational level, No. (%)				<0.01
≤9 years	369 (20.4)	246 (36.6)	123 (10.8)	
>9 years	1,443 (79.6)	426 (63.4)	1,017 (89.2)	
Length of working experience, No. (%)				<0.01
≤3 years	408 (22.5)	257 (38.2)	151 (13.2)	
3–6 years	394 (21.7)	182 (27.1)	212 (18.6)	
>6 years	1,010 (55.7)	233 (34.7)	777 (68.2)	
Frequent night shift (>1 day per week), No. (%)				0.22
Yes	1,266 (69.9)	481 (71.6)	785 (68.9)	
No	546 (30.1)	191 (28.4)	355 (31.1)	
Having organic diseases, No. (%)				<0.01
Yes	126 (7.0)	19 (2.8)	107 (9.4)	
No	1,686 (93.0)	653 (97.2)	1,033 (90.6)	

“*” Chi-square or Fisher exact test for categorical variables and independent-sample non-parametric Mann-Whitney *U* test for abnormal distributed continuous variables.

“^#^” Married category included widowed and divorced participants.

LTCFs, Long-Term Care Facilities.

### Scores and severity of measurements

Compared with patient caregivers in hospitals’ group, the caregivers of older adults in LTCFs’ group showed lower total scale scores assessing symptoms of anxiety and depression, and perceived stress [median (IQR) GAD-7 total score 2.0 (0.0–7.0) vs. 4.0 (1.0–7.0), *p* < 0.01; median (IQR) PHQ-9 total score 2.0 (0.0–7.0) vs. 4.0 (1.0–9.0), *p* < 0.01; median (IQR) PSS-14 total score 21.0 (14.0–28.0) vs. 24.0 (16.0–28.0), *p* < 0.01], as well as higher total score on the wellbeing scale [median (IQR) WHO-5 total score 20.0 (14.0–24.0) vs. 18.0 (10.0–20.0), *p* < 0.01] as shown in Table 2. However, the caregivers of older adults in LTCFs and patient caregivers in hospitals reported similar total score on scale assessing symptoms of insomnia [median (IQR) ISI total score 7.0 (2.0–12.0) vs. 8.0 (3.0–13.0), *p* = 0.22].

**TABLE 2:** Scores of insomnia, anxiety, depression, wellbeing, and stress measurements in total group and subgroups.

Scale and score	Median (IQR)			*P*-values*
	Total (*n* = 1,812)	Caregivers of older adults in LTCFs (*n* = 672)	Patient caregivers in hospitals (*n* = 1,140)	
ISI total score	7.0 (3.0–12.5)	7.0 (2.0–12.0)	8.0 (3.0–13.0)	0.22
GAD-7 total score	4.0 (0–7.0)	2.0 (0–7.0)	4.0 (1.0–7.0)	<0.01
PHQ-9 total score	4.0 (0–8.0)	2.0 (0–7.0)	4.0 (1.0–9.0)	<0.01
WHO-5 total score	19.0 (11.0–21.0)	20.0 (14.0–24.0)	18.0 (10.0–20.0)	<0.01
PSS-14 total score	23.0 (15.0–28.0)	21.0 (14.0–28.0)	24.0 (16.0–28.0)	<0.01

“*” Independent-sample non-parametric Mann-Whitney *U* test.

LTCFs, Long-Term Care Facilities; ISI, Insomnia Severity Index; GAD-7, Generalized Anxiety Disorder-7; PHQ-9, Patient Health Questionnaire-9; WHO-5, The 5-item World Health Organization Wellbeing Index; PSS-14, Perceived Stress Scale-14 item.

As presented in Table 3, a considerable proportion of responders in LTCFs caregivers of older adults’ group had symptoms of insomnia [47.5% (319)], anxiety [36.5% (245)] and depression [36.3% (244)]. Compared with patient caregivers in hospitals, the caregivers of older adults in LTCFs showed similar rates of moderate (14.3 vs. 13.8%, *p* > 0.05) and severe (3.4 vs. 2.3%, *p* > 0.05) insomnia symptoms. Significantly, the caregivers of older adults in LTCFs reported experiencing more symptoms of severe depression (2.8 vs. 1.0%, *p* < 0.05), similar symptoms of moderate (5.4 vs. 6.0%, *p* > 0.05), moderate to severe (2.5 vs. 3.3%, *p* > 0.05) and severe (1.6 vs. 1.1%, *p* > 0.05) anxiety compared with patient caregivers in hospitals. Nevertheless, the older adult caregivers in LTCFs reported lower rates of impaired wellbeing (24.3 vs. 34.3%, *p* < 0.05) and health risk stress (34.2 vs. 42.6%, *p* < 0.05) compared with patient caregivers in hospitals.

**TABLE 3:** Severity categories of insomnia, anxiety, depression, wellbeing and stress measurements in total group and subgroups.

Characteristics	Total (*n* = 1,812)	Caregivers of older adults in LTCFs (*n* = 672)	Patient caregivers in hospitals (*n* = 1,140)	*P*-values*
ISI, insomnia symptoms, No. (%)				0.12
Normal	917 (50.6)	353 (52.5)_*a*_	564 (49.5)a	
Subthreshold insomnia	593 (32.7)	200 (29.8)_*a*_	393 (34.5)_*b*_	
Moderate insomnia	253 (14.0)	96 (14.3)_*a*_	157 (13.8)_*a*_	
Severe insomnia	49 (2.7)	23 (3.4)_*a*_	26 (2.3)_*a*_	
GAD-7, anxiety symptoms, No. (%)				<0.01
Normal	1,009 (55.7)	427 (63.5)_*a*_	582 (51.1)_*b*_	
Mild anxiety	620 (34.2)	181 (26.9)_*a*_	439 (38.5)_*b*_	
Moderate anxiety	104 (5.7)	36 (5.4)_*a*_	68 (6.0)_*a*_	
Moderate to severe anxiety	55 (3.0)	17 (2.5)_*a*_	38 (3.3)_*a*_	
Severe anxiety	24 (1.3)	11 (1.6)_*a*_	13 (1.1)_*a*_	
PHQ-9, depression symptoms, No. (%)				<0.01
Normal	1,000 (55.2)	428 (63.7)_*a*_	572 (50.2)_*b*_	
Mild depression	578 (31.9)	174 (25.9)_*a*_	404 (35.4)_*b*_	
Moderate depression	155 (8.6)	39 (5.8)_*a*_	116 (10.2)_*b*_	
Moderate to severe depression	49 (2.7)	12 (1.8)_*a*_	37 (3.2)_*a*_	
Severe depression	30 (1.7)	19 (2.8)_*a*_	11 (1.0)_*b*_	
WHO-5, wellbeing, No. (%)				<0.01
Unimpaired wellbeing	1,258 (69.4)	509 (75.7)_*a*_	749 (65.7)_*b*_	
Impaired wellbeing	554 (30.6)	163 (24.3)_*a*_	391 (34.3)_*b*_	
PSS-14, stress, No. (%)				<0.01
Normal stress	1,096 (60.5)	442 (65.8)_*a*_	664 (54.0)_*b*_	
Health risk stress	716 (39.5)	230 (34.2)_*a*_	486 (42.6)_*b*_	

“*” Chi-square test for categorical variables; Each subscript letter denotes a subset of group categories whose column proportions do not differ significantly from each other at the *p* = 0.05 level.

LTCFs, Long-Term Care Facilities; ISI, Insomnia Severity Index; GAD-7, Generalized Anxiety Disorder-7; PHQ-9, Patient Health Questionnaire-9; WHO-5, The 5-item World Health Organization Wellbeing Index; PSS-14, Perceived Stress Scale-14 item.

### Risk factors of mental health outcomes among caregivers of older adults in long-term care facilities

Risk factors for symptoms of insomnia, anxiety, depression, wellbeing and stress among LTCFs caregivers of older adults identified by multivariate logistic regression analysis is shown in Table 4. For caregivers of older adults in LTCFs, unmarried status was a potent risk factor for all mental health outcomes except depression after adjustment [insomnia, odds ratio (OR) 3.64; 95% confidential interval (CI), 1.94–6.82; *P* < 0.01; anxiety, OR 2.90; 95% CI, 1.34–6.28; *P* < 0.01;impaired wellbeing, OR 1.91; 95% CI, 1.15–3.17; *P* = 0.01; health risk stress, OR 2.61; 95% CI, 1.62–4.19; *P* < 0.01]. Frequent night shift beyond 1 day per week was associated with higher risks of insomnia (OR, 1.93; 95% CI, 1.07–3.47; *P* = 0.02), depression (OR, 2.56; 95% CI, 1.21–5.44; *P* = 0.01) and impaired wellbeing (OR, 1.74; 95% CI, 1.09–2.76; *P* = 0.01). Compared with caregivers with no organic disease, having organic disease appeared to be an independent risk factor for insomnia (OR, 3.89; 95% CI, 1.37–11.01; *P* = 0.01). Likewise, inferior economic condition (income < expenditure) was associated with insomnia (OR, 1.87; 95% CI, 1.10–3.18; *P* = 0.02), depression (OR, 1.98; 95% CI, 1.04–3.75; *P* = 0.03) and health risk stress (OR, 1.76; 95% CI, 1.14–2.70; *P* = 0.01). In addition, being nurses appeared to be an independent predictor of depression (OR, 6.63; 95% CI, 2.92–15.00; *P* < 0.01), lower wellbeing (OR, 3.45; 95% CI, 2.18–5.47; *P* < 0.05) and health risk stress (OR, 2.59; 95% CI, 1.71–3.91; *P* < 0.01).

**TABLE 4:** Risk factors identified by multivariable logistic regression among caregivers of older adults in LTCFs.

Variables	OR (95%CI)	*P*-value
**Model for insomnia**		
Marital status (unmarried vs. married [reference])	3.64 (1.94, 6.82)	<0.01
Economic conditions (income < expenditure vs. income ≥ expenditure [reference])	1.87 (1.10, 3.18)	0.02
Frequent night shift (>1 day per week) (yes vs. no [reference])	1.93 (1.07, 3.47)	0.02
Having organic diseases (yes vs. no [reference])	3.89 (1.37, 11.01)	0.01
**Model for anxiety**		
Marital status (unmarried vs. married [reference])	2.90 (1.34, 6.28)	<0.01
**Model for depression**		
Economic conditions (income < expenditure vs. income ≥ expenditure [reference])	1.98 (1.04, 3.75)	0.03
Frequent night shift (>1 day per week) (yes vs. no [reference])	2.56 (1.21, 5.44)	0.01
Profession (nurses vs. nursing assistants [reference])	6.63 (2.92, 15.00)	<0.01
**Model for wellbeing**		
Marital status (unmarried vs. married [reference])	1.91 (1.15, 3.17)	0.01
Frequent night shift (>1 day per week) (yes vs. no [reference])	1.74 (1.09, 2.76)	0.01
Profession (nurses vs. nursing assistants [reference])	3.45 (2.18, 5.47)	<0.01
**Model for stress**		
Marital status (unmarried vs. married [reference])	2.61 (1.62, 4.19)	<0.01
Economic conditions (income < expenditure vs. income ≥ expenditure [reference])	1.76 (1.14, 2.70)	0.01
Profession (nurses vs. nursing assistants [reference])	2.59 (1.71, 3.91)	<0.01

LTCFs, Long-Term Care Facilities; OR, odds ratio; CI, confidence interval.

## Discussion

Previous researches showed that quarantine during major infectious disease outbreaks was often associated with a negative psychological effect (18). Unsurprisingly, COVID-19 lock-down with obligatory social distancing measures had made negative effects on the mental health of the general public (44). To our knowledge, few published studies have focused on the nursing home staff being strictly closed managed for the aim of protecting the older adults from COVID-19 infection in China. Our study results showed that caregivers of older adults in LTCFs under strict closed management setting during the COVID-19 post-epidemic era had high prevalence of insomnia (47.5%), anxiety (36.5%), depression (36.3%). Compared with old adult caregivers under routine management setting, which reported 19.4–29.8% depression and 44.0–46.8% anxiety (14, 45), old adult caregivers in current study reported higher depression and lower anxiety rate. This may be accounted for the uncertain closed management duration, being away from family members, restriction of liberty, but reduced risk of COVID-19 infection (18). Li et al. found prevalence rate of sleep problems among old adult caregivers was 10.8%, which was much lower than our result during the COVID-19 epidemic (45). Factors, such as anxiety, extreme tiredness, health concerns, social isolation, parenting challenges, and significant behavior changes under strict closed management, could lead to undoing routines and broken circadian rhythms, affecting three sleep regulatory processes of the homeostatic sleep drive, the circadian rhythm, and the arousal system (46). Interestingly, the results showed caregivers in LTCFs reported a lower total score on perceived stress than patient caregivers in hospitals. It was probably because the mean age (41.0 ± 12.4) of caregivers in LTCFs, which stage of life is corresponding with less family pressure and richer life experience (47), was older than patient caregivers in hospitals (36.3 ± 9.0), and the COVID-19 infection risk under closed management was lower than community. Many researches to date have focused on the mental health of healthcare workers, mainly including physicians and nurses, in hospital setting during the initial stage of COVID-19 epidemic (48–50). Little is known about the mental health of total patient caregiver population, nurses and nurse assistants, in hospital setting during the COVID-19 post-epidemic era. The present study investigated the psychological health of patient caregivers in hospital setting and took it as a comparison group to better understand the mental health levels of caregivers of older adults in LTCFs setting which were long been overlooked (51). As the results showed, caregivers of older adults in LTCFs under strict closed management setting reported experiencing more severe symptom of depression, similar symptoms of insomnia and moderate to severe anxiety compared with patient caregivers in hospital setting. The result perhaps can be accounted by heavy health care burden, the long-term dependence of disabled older adults and social isolation during closed management in LTCFs during pandemic (52).

Our research also identified that unmarried status, being nurses, having organic disease, frequent night shift and inferior economic condition were the potential risk factors for developing symptoms of insomnia, anxiety, depression, lower wellbeing and health risk stress in LTCFs caregivers. Married caregivers were less vulnerable to psychological problems because of the strong relationship support from the family (44, 53). Nurses were more likely to develop psychological problems of depression, lower wellbeing and health risk stress than nursing assistant since they have more responsibilities to dealing with emergent medical problems of the frail older adults (54). Caregivers of older adults having organic disease would be less likely to take advantage of their daily work and were more vulnerable to insomnia (35). Previous study showed shift work was associated with increased risk of poor mental health, particularly depressive symptoms (55). Frequent night shift was the risk factor for caregivers of older adults to develop insomnia, depression and lower wellbeing. The long-term dependence of frail older adults 24 h per day and 7 days per week and insufficient number of caregivers made it common for the caregivers of older adults to take frequent night shift (>1 day per week), especially under closed management (54). Similarly with the previous study about association between economic conditions and poor mental health (56), inferior economic condition was associated with high risk of insomnia, depression and health risk stress among caregivers of older adults in LTCFs. Increasing the income of caregivers of older adults seem to be beneficial to improve their mental health outcome. During the COVID-19 post-epidemic era, the LTCFs for older adults will face more challenges (9, 17).

As the main front-line workers caring for the frail older adults, the caregivers of older adults in LTCFs should not be neglected. Our study result showed the caregivers of older adults in LTCFs developed similar severe psychological symptoms than patient caregivers in hospitals. Several risk factors for poor mental health of caregivers of older adults in LTCFs were also found. Intervention measures for these risk factors may help improve the mental health of front-line workers in LTCFs. The results of this research can be used to develop intervention measures for LTCF managers and the government. The findings remind the managers that they need to pay attention to front-line nurses and caregivers with organic disease, allocate human resources and arrange shift work reasonably, provide social support for unmarried caregivers, strengthen humanistic care, give financial rewards, offer digital psychological interventions and shorten the closure management time as possible (18, 42, 54, 57). All of this should be done to improve the mental health status among adult caregivers in LTCFs, thus improving care of old adults and ensuring its sustainable development during COVID-19 pandemic.

### Limitations

This study has several limitations. First, as a cross-sectional study, mental health outcomes of the caregivers of older adults in LTCFs during the COVID-19 closed management progression are uncertain. An investigation of the psychological changes in different period of close-off management would provide a better understanding. Second, the data was collected from an online self-assessments survey, which is bound to some bias. Third, although the sample size of the current study is adequate, the representative of the sample had certain deficiency due to the convenience sampling method. Forth, some potential variables related to mental health status, such as personality tendency, stressful life event and family/social support, have not been examined in detail in the current study.

## Conclusion

In this survey study of caregivers of older adults in LTCFs within Shandong Province during COVID-19 post-epidemic era, a higher prevalence of psychological symptoms was found as well as risk factors for them. Special attention should be paid to promote mental health of caregivers of older adults in LTCFs, particularly those who are unmarried, nurses and having organic disease, have frequent night shift and inferior economic condition.

## Data availability statement

The original contributions presented in this study are included in the article/supplementary material, further inquiries can be directed to the corresponding author/s.

## Ethics statement

The studies involving human participants were reviewed and approved by the Medical Ethics Committee of The Second Hospital of Shandong University. The patients/participants provided their written informed consent to participate in this study.

## Author contributions

QZ: conception and design and critical revision of the manuscript for important intellectual content. HC, YC, YL, HD, BK, and QZ: conduction and administrative, technical, and material support. HC and YL: statistical analysis. HC: drafting of the manuscript. All authors read and approved the final manuscript.

## Footnotes

1
https://www.wjx.cn/m/155311542.aspx
